# Highly Pathogenic Avian Influenza Viruses at the Wild–Domestic Bird Interface in Europe: Future Directions for Research and Surveillance

**DOI:** 10.3390/v13020212

**Published:** 2021-01-30

**Authors:** Josanne H. Verhagen, Ron A. M. Fouchier, Nicola Lewis

**Affiliations:** 1 Department of Viroscience, Erasmus Medical Center, 3015 GD Rotterdam, Zuid-Holland, The Netherlands; j.h.verhagen@erasmusmc.nl (J.H.V.); r.fouchier@erasmusmc.nl (R.A.M.F.); 2 Department of Pathobiology and Population Sciences, The Royal Veterinary College, Hatfield AL9 7TA, Hertfordshire, UK

**Keywords:** avian influenza, migratory birds, highly pathogenic avian influenza, aquatic birds, migratory flyways, influenza surveillance, influenza subtypes, evolution, epidemiology, ecology

## Abstract

Highly pathogenic avian influenza (HPAI) outbreaks in wild birds and poultry are no longer a rare phenomenon in Europe. In the past 15 years, HPAI outbreaks—in particular those caused by H5 viruses derived from the A/Goose/Guangdong/1/1996 lineage that emerged in southeast Asia in 1996—have been occuring with increasing frequency in Europe. Between 2005 and 2020, at least ten HPAI H5 incursions were identified in Europe resulting in mass mortalities among poultry and wild birds. Until 2009, the HPAI H5 virus outbreaks in Europe were caused by HPAI H5N1 clade 2.2 viruses, while from 2014 onwards HPAI H5 clade 2.3.4.4 viruses dominated outbreaks, with abundant genetic reassortments yielding subtypes H5N1, H5N2, H5N3, H5N4, H5N5, H5N6 and H5N8. The majority of HPAI H5 virus detections in wild and domestic birds within Europe coincide with southwest/westward fall migration and large local waterbird aggregations during wintering. In this review we provide an overview of HPAI H5 virus epidemiology, ecology and evolution at the interface between poultry and wild birds based on 15 years of avian influenza virus surveillance in Europe, and assess future directions for HPAI virus research and surveillance, including the integration of whole genome sequencing, host identification and avian ecology into risk-based surveillance and analyses.

## 1. Introduction

Wild waterbirds belonging to the order Anseriformes (mainly ducks, geese, swans) and Charadriiformes (gulls, terns, shorebirds) are the natural reservoir of low pathogenic avian influenza (LPAI) viruses. From wild waterbirds, LPAI viruses can be transmitted directly or indirectly to domestic birds, other wild animals, domestic animals, and humans [1]. LPAI viruses can cause mild to severe disease upon transmission to poultry (“spillover”). LPAI viruses of the subtypes H5 and H7 can evolve into highly pathogenic avian influenza (HPAI) viruses upon introduction into poultry, causing severe disease and mortality in poultry, in particular in birds of the order Galliformes (mainly chickens, turkeys) [2]. From poultry, avian influenza viruses can be transmitted to wild birds (“spillback”) in which the viruses can circulate asymptomatically, or cause disease and mortality [3,4]. The evolution and epidemiology of viruses is a consequence of the interplay among the viruses, their hosts and their environments [3]. For LPAI and HPAI viruses, an important component of virus evolution and epidemiology occurs at the wild–domestic interface. In Europe, between 2000 and 2018, poultry production has almost doubled [5], whilst common European wild birds have declined [6]. In the past 20 years, HPAI viruses have been detected more abundantly in domestic and, in particular, in wild birds in Europe than before the emergence of HPAI H5 viruses of the A/Goose/Guangdong/1/1996 (GsGd) lineage in southeast Asia [2,4,7]. The recurring HPAI H5 virus incursions raise questions about the mechanisms of the novel emergence of HPAI H5 viruses, including source, routes of introduction into Europe and into poultry farms.

Until 1997, HPAI viruses were largely confined to poultry, causing outbreaks of varying sizes that could be contained through the killing of infected poultry, preventive culling of poultry and/or vaccination. HPAI viruses had been detected once in wild birds, but this was not associated with poultry outbreaks [8]. Hence, prior to, during or after poultry outbreaks, no HPAI viruses have been detected in wild birds. However, this changed with the emergence of HPAI H5 GsGd viruses. HPAI H5 GsGd viruses cause severe and widespread poultry outbreaks with enormous economic losses and were shown to be able to infect a broad range of both bird species and mammals, including humans. Since 2003, HPAI H5 GsGd viruses have become enzootic in multiple countries in south and southeast Asia [9]. During the initial circulation and spread of the HPAI H5 GsGd viruses, the haemagglutinin (HA) genes diversified into multiple genetic lineages (“clades”) [10,11], without evidence of gene exchange between the influenza viruses [12]. Yet, this evolutionary trajectory changed from 2009 onward, when HPAI viruses of subtypes H5N2, H5N3, H5N4, H5N5, H5N6, and H5N8 were found to contain the H5 gene of the GsGd lineage, together with the neuraminidase (NA) and various other genes of LPAI virus origin [13,14,15,16,17]. In particular, HPAI H5 viruses from clade 2.3.4.4 dominated European outbreaks from 2014 onwards, with new genetic assortments and different epidemiological characteristics, such as the wide range of wild bird species affected and HPAI H5 virus incursions in north-western Europe [17,18].

The first HPAI H5 mass mortality event in wild birds was observed in 2005 at Qinghai Lake in China (clade 2.2) [19]. This outbreak was followed within a few months by outbreaks among wild birds and poultry within Asia, and subsequently through Siberia to the Middle East, Europe and Africa. The initial European H5 virus incursions in 2005 marked the start of a series of virus introductions into Europe. In 2009, a new clade of HPAI H5 GsGd caused mortality among wild birds at Qinghai Lake (clade 2.3.2), followed by outbreaks among poultry and wild birds within Asia. These viruses were ancestral to the HPAI H5 clade 2.3.2.1c viruses detected in eastern Europe in 2010 and 2015. From 2014 onwards, another clade of HPAI H5 GsGd (clade 2.3.4.4) caused several outbreaks among wild birds in Southeast Asia, followed by spread through Siberia to the Middle East, Europe, Africa, and North America [20]. The spillback of HPAI H5 GsGd viruses from poultry into wild birds and its spread throughout Asia, the Middle East, Africa, Europe and North America has put a focus on the role of wild birds in the geographical spread of HPAI H5 GsGd viruses [17,18,21].

In response to the emergence of HPAI H5 GsGd viruses, outbreaks in wild birds and the long-distance dispersal of the viruses, avian influenza virus surveillance programs in wild birds and poultry were initiated and intensified globally. These programs aimed at the early detection of (novel) HPAI H5 viruses into new geographical regions, and to investigate the role of wild birds in the spread of these HPAI H5 viruses. In this review, we give an overview of HPAI virus epidemiology, ecology and evolution at the interface between poultry and wild birds based on 15 years of avian influenza virus surveillance in Europe. Significant progress has been made in describing HPAI H5 virus evolution and epidemiology—in particular, with respect to the identification of host species, time periods, habitats and geographies associated with increased risks of HPAI H5 introduction—due to collaborative efforts of virologists, ornithologists, ecologists, pathologists, and mathematical modellers amongst others, and due to faster and specific diagnostics, in particular, gene sequencing technologies. Lastly, we provide recommendations for future research and surveillance to achieve earlier detection of novel HPAI viruses and further understanding of HPAI H5 evolution and epidemiology, as major gaps in knowledge remain.

## 2. Recurring Emergence of HPAI H5 Viruses in Europe 2005–2020

Until 2005, no spillback events from HPAI viruses from poultry into wild birds were observed in Europe. HPAI viruses have been described to cause outbreaks in poultry in Europe since the first description of the disease in Italy in 1878, which was followed by epizootics until 1935 [22]. Next, local HPAI virus outbreaks in poultry in Europe were reported in Scotland in 1959, England in 1962, Germany in 1979, England in 1979, Ireland in 1983, England in 1991, Italy in 1997, England in 2008, Spain in 2009, Italy in 2013, Germany and England in 2015, and Italy in 2016 [2,23,24]. In contrast to these local outbreaks, large HPAI outbreaks were seen in Italy in 1999, the Netherlands in 2003 and France in 2015–2016, involving the culling of millions of birds. Of these, the HPAI H7 outbreaks in Italy [25] and in the Netherlands [26,27] were directly preceded by the circulation of LPAI viruses in poultry. Furthermore, through wild bird surveillance programs, LPAI ancestral viruses were identified of the HPAI viruses causing outbreaks in Europe between 1997–2003 and 2015–2016 [28,29,30]. Thus, wild birds play a role in avian influenza virus (AIV) epidemiology by donating LPAI viruses to poultry, which can subsequently result in outbreaks largely limited to poultry; however, this changed with the emergence of HPAI H5 GsGd viruses that have caused outbreaks in Europe since 2005. Between 2005 and 2020, at least ten incursions of HPAI H5 GsGd viruses were observed in Europe (i.e., clade 2.2 *n* = 3, clade 2.3.2.1c *n* = 2, clade 2.3.4.4 *n* = 5).

### 2.1. H5 Clade 2.2 (2005–2009)

In July and August 2005, HPAI H5 clade 2.2 viruses were detected that caused mortality at poultry farms in Russia in Western Siberia (Novosibirsk region) and Kazakhstan, and during mass die-offs of migratory ducks, geese and swans in Mongolia in August 2005. The viruses were genetically closely related to the viruses detected during wild bird mass mortality in 2005 at Qinghai Lake in China. From July onwards, HPAI H5 viruses kept causing outbreaks among poultry farms and viruses were detected in wild birds in Russia [24,31]. 

**Figure 1:**
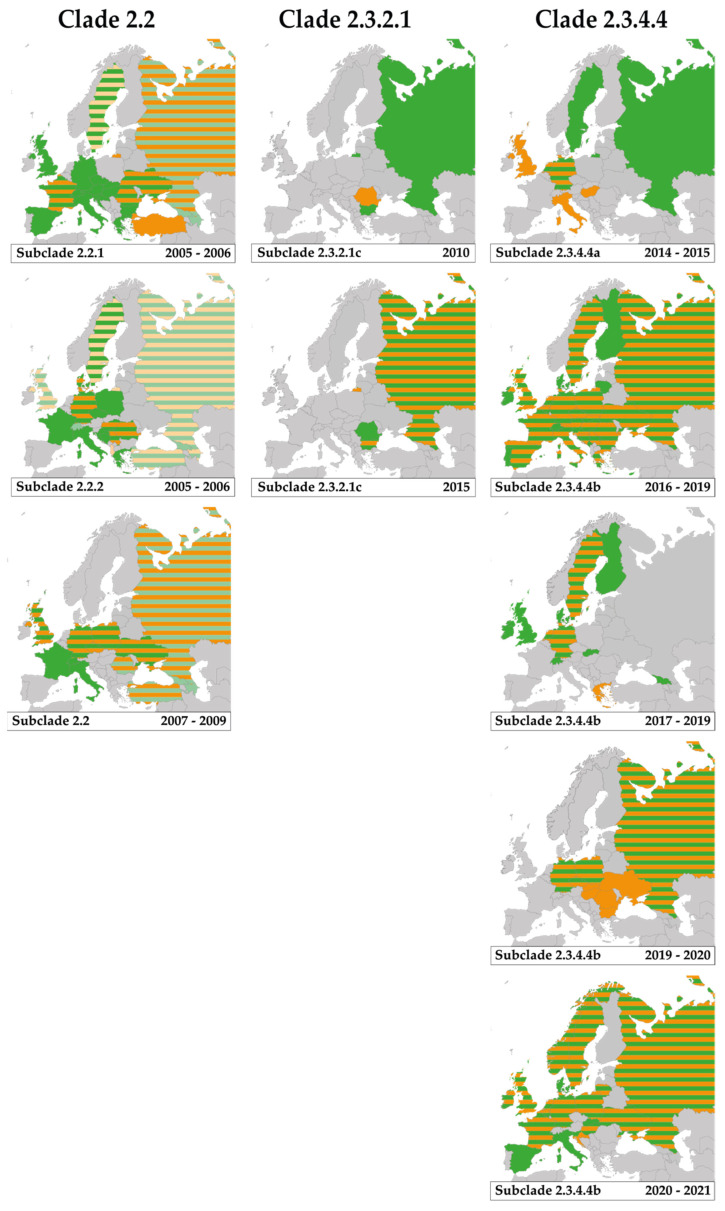
Highly pathogenic avian influenza H5 GsGd virus detections in wild birds and poultry in Europe, 2005–2020. Each panel shows the European countries in which the subclade had been detected, including the time period. Green, viruses detected in wild birds; orange, viruses detected in poultry; green-orange striped pattern, subclade detected in both wild birds and poultry at country level; light green, clade 2.2 viruses detected in wild birds and subclade unknown; light orange, clade 2.2 viruses detected in poultry and subclade unknown. References used [24,34,35,37,38,39,40,41,42,43,44,45,46,47,48,49,50,51,52,53,54,55,56,57].

In October 2005, the first HPAI H5N1 viruses were detected in southeast Europe. In western Turkey during mortality at a backyard farm (clade 2.2.1) [7,24], in the Danube delta in Romania in wild birds (i.e., gray heron (*Ardea cinerea*) and mute swans (*Cygnus olor*)) and during mortality in backyard farms (clade 2.2.1 and 2.2.2) [24,32,33], and in Croatia in mute swans (clade 2.2.2) [24,34]. Within Europe, clade 2.2.1 viruses have been detected in at least 19 countries and clade 2.2.2 viruses have been detected in at least 13 countries (Table 1, Figure 1). Clade 2.2.1 and 2.2.2 were for the last time detected in Europe in 2006.

Highly pathogenic avian influenza H5N1 clade 2.2 viruses were first detected in Italy in a mute swan in October 2006 (based on the isolate A/Cygnus olor/742/2006 in e.g., [7]), followed by detections in 2007 in at least 12 European countries (Table 1, Figure 1). H5 clade 2.2 viruses were for the last time detected in a common pochard (*Aythya ferina*) in Europe in 2009 [35]. Previously, clade 2.2 viruses were detected in Russia (Siberia), Mongolia in 2006 [36]. Between 2005 and 2009, HPAI H5 clade 2.2 viruses (i.e., clade 2.2.1, 2.2.2 and 2.2) were detected in wild birds in 25 European countries, while HPAI H5 viruses were detected in poultry in 16 European countries (Table 1). Genetic analyses, based on HPAI H5 clade 2.2 viruses pointed to at least three virus introductions into Europe [35,37].

### 2.2. H5 Clade 2.3.2.1 (2010–2015)

A novel HPAI H5 virus of clade 2.3.2.1c caused two outbreaks among backyard poultry in Romania in March 2010 [58]. In the same month, HPAI H5N1 clade 2.3.2.1 viruses were isolated from a common buzzard (*Buteo buteo*) found dead in Bulgaria [59]. Prior to the detection of clade 2.3.2.1c in Bulgaria and Romania, clade 2.3.2 was detected in 2009 in wild birds in Mongolia and Russia (Siberia). First, in whooper swans (*Cygnus cygnus*) in Mongolia in May 2009 [60], followed by detection in great-crested grebe (*Podiceps cristatus*), little grebe (*Tachybaptus ruficollis*), black-headed gull (*Chroicocephalus ridibundus*) and Eurasian spoonbill (*Platalea leucorodia*) at Uvs-Nuur Lake, Tyva Republic in Russia (at the border with Mongolia) in June 2009 [60]. Viruses belonging to the same genetic clade 2.3.2.1c were again detected in whooper swans and graylag geese (*Anser anser*) in Sukhbaatar, Mongolia, in May 2010, and in great-crested grebe, goosander (*Mergus merganser*), gray heron, gadwall (*Mareca strepera*) and Eurasian spoonbill in Tyva, Russia, in July 2010 [61]. The clade 2.3.2.1 viruses were genetically closely related to H5N1 clade 2.3.2 detected in village poultry in the Russian Far East in April 2008 [36]. In March 2015, the HPAI H5 clade 2.3.2.1c again caused outbreaks in wild birds (i.e., Dalmatian pelicans (*Pelecanus crispus*), rock pigeon (*Columba livea*), black-headed gull) and backyard poultry in Bulgaria and in wild birds (i.e., Dalmatian pelicans) in Romania [24,62,63]. No H5 clade 2.3.2.1c viruses were detected in Europe since 2015.

### 2.3. H5 Clade 2.3.4.4 (2014–2020)

Since 2013, ancestral HPAI H5 viruses of the clade 2.3.4.4 in combination with different neuraminidase subtypes (e.g., H5N1, H5N6, H5N8) have been circulating in Southeast Asia. Clade 2.3.4.4 of two distinct genetic groups (i.e., group A or Buan-like and group B or Gochang-like) were first detected in China and South Korea in late 2013/early 2014 [64,65]. Group A viruses (i.e., clade 2.3.4.4a) emerged in late 2014 and spread to Europe and North America simultaneously. Group B (i.e., clade 2.3.4.4b) viruses were not reported anywhere until detected again at Qinghai Lake in China and at Lake Uvs-Nuur in Russia in May 2016 [66,67] and subsequently spread to Europe.

In November 2014, HPAI H5N8 clade 2.3.4.4a were first detected in wild birds and poultry in Europe. First in a turkey holding in northeast Germany [68], shortly followed by detections in farms in the Netherlands [69] and the UK [70]. Additionally, genetically closely related viruses were detected in apparently healthy Eurasian teal (*Anas crecca*) in Germany [68] and Eurasian wigeons (*Mareca penelope*) in the Netherlands in November 2014 [56]. These detections were followed by clade 2.3.4.4a virus detections in a turkey holding in Italy in December 2014, in domestic ducks in Hungary in February 2015 [24], and in mute swans found dead in Sweden in February and March 2015 [71] (Table 1, Figure 1). After March 2015 no clade 2.3.4.4a viruses have been detected in Europe. Prior to detection in Europe, HPAI H5N8 clade 2.3.4.4a viruses—with the same genome constellation as the viruses detected in poultry and wild birds in Europe in 2014–2015—were detected in September 2014 in a Eurasian wigeon in eastern Russia [35,72]. 

**Figure 2:**
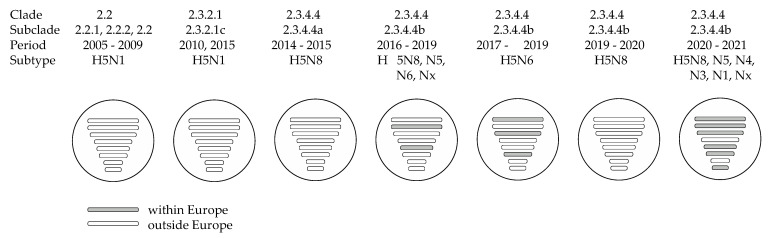
Highly pathogenic avian influenza H5 GsGd virus incursions into Europe (2005–2020) were associated with abundant reassortment with Asian and European low pathogenic avian influenza viruses. Multiple different clade 2.3.4.4b assortments circulated with some variants containing genomic segments derived from low pathogenic avian influenza viruses circulating in wild birds in Europe, suggesting reassortment upon introduction into Europe. Please note that inference of reassortment location is heavily biased by the intensity of influenza surveillance and the availability of virus genome sequences. References used [35,55,76].

Late October 2016, newly introduced HPAI H5 clade 2.3.4.4b viruses were isolated from poultry in Hungary, followed by the detections in tufted ducks (*Aythya fuligula*) in Germany [73]. In total, HPAI H5 clade 2.3.4.4b viruses were detected in at least 31 European countries [24]. Following the initial detection of H5N8 clade 2.3.4.4b viruses, abundant reassortment with LPAI viruses was observed in poultry and wild birds in 2016–2017, resulting in the emergence of HPAI H5N5 viruses in several European countries between November 2016 and June 2017 and HPAI H5N6 viruses in Greece in February in 2017 [24,35,57,73,74,75,76,77,78,79]. The HA, M and NS of H5 clade 2.3.4.4b viruses were derived from HPAI H5N8 viruses as detected in Russia and Mongolia in 2016, while the remaining segments of the reassortants were genetically closely related to LPAI viruses in Central Asia (Russia, China, Mongolia), East Asia (Japan), and Europe (i.e., PB1 and NS genes) [35,76] (Figure 2). Viruses of H5N8 clade 2.3.4.4b detected in Russia in wild birds in May–June 2016 prior to detection in Europe [66,80], had the HA, NA, NS from clade 2.3.4.4b HPAI viruses and PB2, PB1, PA, NP and M from Eurasian (Mongolia, Vietnam) LPAI viruses [66]. These detections were followed by poultry outbreaks in Russia in autumn and winter 2016–2017 and the emergence of reassortant viruses H5N2 and H5N5 [81], which resulted in the culling of 2.6 million poultry in Russia [82].

From December 2017 onwards, HPAI clade 2.3.4.4b H5N6 viruses with a novel gene constellation were detected in Europe. Early December HPAI H5N6 viruses were detected at a duck farm in the Netherlands and in wild birds (i.e., mute swan, tufted duck) [79] and in a common pochard (*Aythya ferina*) in Germany [35]. Including these initial detections in northwest Europe, H5N6 viruses were detected in at least 11 European countries (Table 1, Figure 1). The H5N6 clade 2.3.4.4b viruses contained PB1, HA, NP, M and NS genes derived from the 2016–2017 H5N8 viruses, while PB2, PA and NA were genetically closely related to Eurasian wild bird LPAI viruses (Figure 2) [35,57,79,83]. The HPAI H5N6 virus was different from the H5N6 virus associated with human infections in Asia [79]. 

In December 2019 onwards, HPAI H5N8 clade 2.3.4.4b viruses were detected in poultry in Poland. Next, H5N8 viruses were detected in a greater white-fronted goose (*Anser albifrons*) in Germany [75], and in poultry in Romania, Slovakia, Ukraine, Hungary, and Czech in January 2020, and in Bulgaria in February 2020 [24]. The genome was mainly derived from earlier HPAI H5N8 clade 2.3.4.4b viruses, with the PB1 and NP genetically most closely related to LPAI H3N8 viruses circulated in Central Asia (Ural/Siberia, Russia) (Figure 2) [75].

In mid-October 2020, novel HPAI H5N8 viruses—differing from those circulating earlier in 2020—were detected in Eurasian wigeons and mute swans shortly followed by detections in poultry in the Netherlands [84]. From November 2020 onwards HPAI H5 viruses were detected in at least 19 European countries. Simultaneously, multiple HPAI H5 reassortant viruses were detected in Belgium, Denmark, Germany, Ireland, Italy, the Netherlands, Slovenia, Sweden and the UK, with up to six genes replaced with local European strains (i.e., PB2, PB1, PA, NP, NA, NS) (Figure 2) [38,55]. The vast majority of HPAI viruses detected in wild birds and poultry were H5N8 viruses, while H5N1, H5N3, H5N4 and H5N5 were predominantly isolated from wild birds [38]. In addition, additional subtypes may be added as laboratories in multiple countries have reported H5Nx in wild birds [38]. The viruses did not cluster with HPAI H5 clade 2.3.4.4b viruses circulating previously in Europe but may be partially derived from North African/Middle Eastern/South-West Asian strains [55]. Prior to these detections, from August 2020 onwards HPAI H5N5 and H5N8 were detected in poultry in Russia and H5N8 viruses in September 2020 in Kazakhstan and in October 2020 in Israel [24,84].

## 3. Underlying Mechanisms of Emergence of Novel HPAI H5 Viruses at the Wild–Domestic Bird Interface

### 3.1. Virus-Related Drivers of Emergence of Novel HPAI H5 Viruses in Wild/domestic Birds

The reassortment of genomic segments is very common in wild waterbirds and central to the emergence of novel AIVs [85,86]. In wild (water)birds, LPAI viruses form transient genome constellations without the strong selective pressure to be maintained as linked genomes, in contrast to stable genome constellations that characterize the evolution of mammalian-adapted influenza A viruses (IAVs) [85]. The faecal–oral transmission route of LPAI viruses, high AIV diversity in water samples, and the generally low virulence of LPAI viruses in wild waterbirds optimize conditions for co-infections that may lead to genetic reassortment [87]. In addition, internal segments of novel HPAI H5 GsGd viruses have their origin in LPAI H9N2 viruses that circulate widely among poultry in Southeast Asia [20], suggesting a role for poultry as a donor of genomic segments integrated into novel HPAI H5 viruses. The NA genes of the HPAI H5 viruses that emerged in China may have derived from H3N2, H6N6 and H3N8 viruses [20], which are common HA subtypes in waterfowl. Some HPAI H5 GsGd virus introductions into Europe showed stable genome constellations (e.g., [56]). However, since 2016, clade 2.3.4.4b viruses detected in Europe showed abundant reassortment with Eurasian LPAI viruses (e.g., [35,77]) (Figure 2). We hypothesize that the timing of H5 emergence in wild birds in relation to the birds’ annual cycle and LPAI virus peak prevalence (e.g., fall in dabbling ducks in temperate climates), affect genetic reassortment and maintenance of novel HPAI H5 variants.

Despite understanding that HPAI viruses face different selective pressures depending on the host populations the viruses circulate in [88], much is unknown about selective pressures on HPAI H5 viruses. For instance, mechanisms underlying genomic changes including genetic reassortments, in relation to host adaptation (i.e., virus attachment, tissue tropism, replication and release from host species), activation of the HA, infectivity, transmissibility, environmental survival, and immune evasion. Co-infecting viruses need to match with respect to their RNA (e.g., packaging signals) and proteins (e.g., HA–NA interactions) in order to reassort [89]. In addition, the genomic segments PB2, PA, NP, and M may play a pivotal role during genome packaging [90], while packaging signals (i.e., segment- and strain-specific signals compromising of both untranslated region and coding sequence at the 3′ and 5′ end of each segment) may bias or restrict reassortment [91]. So far, findings on RNA–RNA interactions are ambiguous. RNA–RNA interactions may impact the fitness of reassortant progeny [92], yet a recent study suggests that RNA–RNA interactions between influenza genome segments are flexible [93]. The minimal IAV-specific conditions for reassortment are optimal RNA and/or protein interactions (i.e., matching RNA and/or matching proteins) and/or timing and dose of virus, amongst other factors [89]. Based on intra-subtype reassortments, derived from human H3N2 IAVs, it was revealed that reassortment is usually deleterious, resulting in a negative selection of reassortant progeny [89]. The virus-specific conditions for reassortment of HPAI H5 viruses need further clarification.

Most of the mutations described in AIVs isolated from wild birds are related to spillback events of HPAI H5N1 viruses from domestic birds [3]. In domestic (gallinaceous) birds, mutations in genomic segments HA [94], NA [95] and NS [96] have been associated with virulence and/or host switching, while in (wild) ducks, mutations in genomic segments PB1 [97], PA [98] and HA [99] were associated with virulence and/or host change.

The acid stability of HPAI H5 viruses was shown to affect pathogenicity and viral environmental persistence. In chickens, an increase in H5N1 pathogenicity was correlated with an increase in the pH of HA activation (which is needed in order for the virus to bind and infect the host cell), which was linked to variations at residues 104 and 115, located in the N- and C-termini of helix-110 of HA1 [94]. In contrast, in mallards (*Anas platyrhynchos*), an H5N1 virus carrying an H24Q substitution was shed more extensively from infected mallards into drinking water and persisted for a longer period in the environment; however, with a decrease in the pH of HA activation [99]. In an LPAI H11N9 virus, HA amino acid substitutions A198V and S274F were associated with IAV adaptation to chickens [100].

Deletion in the stalk of the NA is seen as an adaptation associated with the transmission of AIVs from aquatic birds to terrestrial poultry. NA stalk deletions favoured replication and enhanced virulence in chickens (19 amino acid deletions [aa del] human HPAI H5N1 [101]), increased virulence in chickens (20 aa del chicken HPAI H5N1 [102]), was a main determinant of respiratory tract infection in chickens (27 aa del wild bird LPAI H2N2 [103]), and increased shedding and virulence in chickens (22 aa del turkey LPAI H7N1 [95]). In contrast to findings in chickens, long-stalk virus showed higher levels of intestinal replication and faecal shedding in Pekin ducks (LPAI H7N1 turkey isolate [95]). Furthermore, based on the LPAI H11N9 virus, substitutions and deletions were observed in NA in chicken only, not in Pekin ducks [104]. 

Genes of the polymerase complex (PB2, PB1, PA) are involved in virulence and host range. For instance, PB1 with the substitution Y436H and PA with the substitution T515A, reduced the virulence of a small-plaque phenotype of A/Vietnam/1203/04 (H5N1), which is known to be highly virulent for ferrets, mice and mallards [97]. The PA, with substitutions S224P and N383D of the A/duck/Hubei/49/05 virus clade 2.3.4, was associated with a highly virulent phenotype in ducks [98]. Moreover, outbreak severity of HPAI H5N8 clade 2.3.4.4a and clade 2.3.4.4b viruses was inversely related to polymerase complex activity [105]. The diversity of polymerase subunits and the relatively high rates of reassortment particularly in ducks [106] mean that assessing the relative virulence of different HPAI H5 viruses in wild hosts—even within Anseriformes—is challenging. In chicken, a five amino acid deletion at 80-84 aa of the NS1 gene—present in most HPAI H5N1 isolates since 2002—combined with D92E of the NS1 gene, has been associated with increased virulence and improved replication [96,107]. 

The comparison of next-generation sequencing (NGS) generated sequences of HPAI H5 isolated from live and dead birds may inform about host-specific mutations and virulence. For instance, a unique pattern of amino acid substitutions in the regions of viral proteins crucial for virulence of H5N1 viruses was observed in an HPAI H5 GsGd virus isolated from a live bird (A/common gull/Chany/P/2006) (i.e., HA 111Y, M2 28A, NS1 101I and 171D, PB2 667I and NS2 15T) [108].

### 3.2. Environmental-Related Drivers for Emergence of Novel HPAI H5 Viruses in Wild/Domestic Birds

The role of the environment on HPAI H5 virus emergence is understudied, as little is known about selective pressures from the environment on the emergence of novel HPAI H5 viruses. However, based on findings with respect to LPAI viruses, the environment plays a role in virus maintenance [109] and the accumulation of virus genomes [87], hereby supporting virus persistence, transmission in particular among wild waterbirds, and co-infections that may lead to genetic reassortment. LPAI viruses can survive in the environment for prolonged periods of time, while the duration of LPAI virus environmental persistence varies by subtype and genotype [87,110,111,112], and is dependent of abiotic factors, such as temperature, salinity, pH, mineral content of the medium [111,112,113,114,115,116]. The longest LPAI virus persistence in samples from the environment was observed for water samples, followed by sediment, surfaces, and air [113]. In addition, LPAI virus cultivation from water samples required minimal viral titres, compared with IAV cultivation from sediment [117]. Furthermore, mathematical models suggest that environmental reservoirs could play an important role in the ecology of LPAI viruses [118,119,120,121]. Similar to LPAI viruses, HPAI viruses survive for a longer period of time at lower temperatures, while high salinity decreased virus survival [122]. Based on laboratory experiments, HPAI viruses do not persist in the environment as long as LPAI viruses [122]. Conversely, HPAI viruses were found to persist longer than LPAI viruses in poultry litter material [123,124]. Thus, virus persistence in the environment is likely to vary among different H5 lineages and habitats and affects the likelihood of virus maintenance within and among wild bird and domestic bird populations.

The abundance, distribution and timing of the migration of wild waterbird populations—and thus the spread of avian pathogens such as HPAI H5 viruses—is partially shaped by environmental factors, such as temperature and habitat availability. For instance, low temperatures during wintering have been associated with aggregations of wild waterbirds and HPAI H5N1 outbreaks [125]. Similarly, it has been hypothesized that the cold winter of 2005–2006 assisted in the spread and persistence of H5N1 influenza viruses in wild birds in Europe [126]. Furthermore, winter weather conditions in western and northern Europe had considerable impact on the waterbird abundance at the breeding grounds in Finland, with the increased breeding abundance of waterbird species after mild winters (likely due to improved bird survival) [127]. Based on long-term ringing and field counting data in northwest Europe (1990–2013), the centre of gravity in the abundance of diving ducks—of which some species (common pochard, tufted duck) have shown to be susceptible to infection with HPAI H5 viruses—showed a steadily shift north-eastwards over the past three decades, while no long-term shifts were shown for dabbling ducks, swans and geese [127]. Furthermore, a long-term study (1979–2009) observed delayed departure from the breeding sites in northern Europe of six (including graylag goose, Eurasian wigeon, Eurasian green-winged teal, tufted duck) out of 15 waterfowl species studied [128]. Habitat characteristics, in particular the availability of wetlands, affect waterbird abundance and distribution [129]. A study based on the tracking data of 386 birds of 36 bird species hypothesizes that as habitat homogenization increases, some bird populations might be forced to travel increasingly longer distances to meet their diverse foraging and reproduction habitats [130].

### 3.3. Host-Related Drivers of Emergence of Novel HPAI H5 Viruses in Wild/Domestic Birds

Wild and domestic birds show different susceptibility to infection and disease of HPAI H5 viruses. Susceptibility to infection and disease has been mostly studied in domestic chickens and (domestic) ducks. For instance, in domestic birds, H5N8 HPAI virus (clade 2.3.4.4a) exposure resulted in less disease among ducks compared with chickens and turkeys [131]. In wild birds, no disease was observed upon H5N8 HPAI virus (clade 2.3.4.4a) exposure of six duck species [132]. Furthermore, mute swans exposed to the HPAI clade 2.2.2 showed variable susceptibility to disease and virus tropism, suggesting within species differences in susceptibility to disease [133]. Susceptibility to infection and disease has also been shown to differ among HPAI H5 virus lineages. For instance, Pekin ducks showed variable susceptibility to disease; exposure to HPAI clade 2.3.4.4a H5N8 2014 resulted in 60% mortality [134], while exposure to HPAI clade 2.3.4.4b H5N8 2016 or H5N6 2017 resulted in 100% mortality [134,135]. Based on field surveillance data, more wild birds died during the circulation of clade 2.3.4.4b H5N8 2016 than during the circulation of clade 2.3.4.4b H5N6 2017 [134]. The difference in susceptibility to infection and disease of birds complicates analyses of HPAI virus emergence as novel viruses can circulate unnoticed in particular in domestic ducks and can be distributed over vast distances by birds that are not included in the passive surveillance of dead birds.

Wild bird species and individual birds play different roles in the epidemiology of HPAI H5 viruses. Wild birds that may act as a vector most successfully are migratory birds that excrete virus in the absence of clinical signs of disease (e.g., in Europe, birds of the species Eurasian wigeon, Eurasian green-winged teal, mallard, black-headed gull) (Table 2). These are common bird species with varying migratory patterns. Eurasian wigeons are typically long-distance migrants with populations wintering in West/Southwest Europe and breeding in areas ranging from North-eastern Europe to far into Russia. Eurasian green-winged teals are medium to long-distance migrants [136], while mallards and black-headed gulls are medium to short-distance migrants and partially sedentary. In contrast, sentinel birds may play a role in local HPAI virus amplification; however, they are expected not to migrate while infected (e.g., in Europe, birds of the species mute swan). Mute swans are medium to short-distance migrants with populations that are mostly sedentary. Prior to the first HPAI GsGd H5 virus introductions into Europe, “higher risk” birds have been identified based on the birds’ ecology and LPAI virus occurrence, resulting in a list of 26 Anseriformes and Charadriiformes species [137,138]. This list was followed by a “target species” list of 50 species in 2010, partly based on HPAI H5 detections in Europe, that could be used to target wild bird surveillance. From 2005 to 2020, HPAI H5 GsGd viruses have been detected in at least 86 bird species sampled in Europe (Table 2). The majority of HPAI H5 viruses have been isolated from birds found dead, yet HPAI H5 viruses have also been isolated from apparently healthy birds of several species (Table 2). In addition to HPAI H5 virus detection, host species have been defined based on serology. The presence of HPAI H5 virus and/or HPAI H5-specific antibodies in live birds suggests that (some individuals of) these bird species can (asymptomatically) carry the virus and act as a vector. Experimental infections in captivity confirmed differences among bird species’ susceptibility to infection and/or disease [132,133,135,139,140,141,142,143]. Given the heterogeneity among individuals in susceptibility to infection and disease, in some cases a single species can act as vector as well as a sentinel [132,133,135,139].

Prior LPAI virus exposure affects the susceptibility to infection and disease of HPAI H5 viruses in wild birds; however, may support HPAI H5 virus spread and maintenance. Mallards pre-exposed to LPAI viruses in a semi-natural setting excreted HPAI H5 clade 2.3.4.4b virus but survived virus challenge [135]. Similarly, pre-exposure of mallards to H4N6 and H5N2 LPAI viruses in an experimental setting resulted in varying excretion of HPAI H5N1 clade 1 (A/duck/Vietnam/TG24-01/05), and less than naïve mallards [143]. Partial protective immunity may facilitate virus spread through increased host survival and thus longer HPAI H5 virus excretion [133,144]. In Europe, poultry is not vaccinated for export reasons, resulting in an enormous susceptible host population for AIVs. It is currently unknown how long a protective effect of prior HPAI H5 virus infection lasts and how this affects HPAI H5 epidemiology. For instance, a lower circulation of HPAI H5N6 viruses in 2017 was observed, compared to HPAI H5N8 in 2016–2017 [134], which may—besides other factors—be related to partial immunity. 

There is limited evidence for pre-existing immunity as selective pressure for HPAI H5 virus evolution in birds, in contrast to IAV evolution in humans, swine and horses. Pre-existing immunity in wild birds has shown to affect subtype distributions [145], yet it is unknown if/how pre-existing immunity facilitates antigenic variation within HA subtypes of viruses isolated from birds. To avoid neutralisation by pre-existing serum antibodies, the antigenic evolution of human influenza A viruses allows escape from immunity induced by earlier strains [146]. In domestic birds, AIV vaccination programs have been linked to gradual antigenic changes in circulating AIVs (“antigenic drift”) [147]. The role of pre-existing (serum) antibodies on HPAI H5 virus evolution in birds has been questioned based on a study on HPAI H5 virus clade 2.1 evolution in poultry in Indonesia [148]. In this study, the antigenic evolution of the viruses did not follow a directional pattern away from earlier viruses; instead antigenic variants were possibly replaced by random newly emerging variants. Potential explanations for this different pattern could be the short lifespan of the majority of poultry (i.e., broiler chicken 5–7 wks) and genetic reassortment with LPAI viruses from free-living (wild) birds, the latter resulting in newly emerging variants. Antibodies raised in Pekin ducks upon HPAI H5N8 clade 2.3.4.4a exposure resulted in similar HI titres with the same clade 2.3.4.4a virus and with H5N8 clade 2.3.4.4b, suggesting no or limited antigenic change [131]. Furthermore, in HPAI H5-specific sera from wild birds sampled in Mongolia and Europe ambiguous patterns with HPAI H5 viruses were observed [149]. In wild birds, some evidence for antigenic variation within HA subtypes is limited to LPAI viruses. For instance, no distinct antigenic variants were identified for H3 or H16 LPAI viruses, while two antigenically different clusters were identified for H13 LPAI viruses [150,151]. In addition, knowledge on the duration of HPAI H5-specific antibody detection in ducks is limited (e.g., 34 days for 9/10 ducks upon virus exposure [152]). To investigate HPAI H5 virus exposure histories of wild birds is challenging, due to waning serum antibody levels and repeated AIV exposures, resulting in low antibody levels and complex hemagglutination inhibition profiles [149]. Additionally, antibody responses to avian influenza viruses in wild birds broaden with age [153]. Thus, a causal relationship between AIV-specific antibody production and virus evolution in wild birds has not been clarified yet for either LPAI [3] or HPAI viruses.

### 3.4. HPAI H5 Virus Introductions into Europe by Wild Birds

The area and timing of HPAI H5 GsGd virus introduction depends on the migration phenology and habitat availability of the host species. Four major entry pathways of HPAI virus via migratory birds into/from the EU were identified: northeast, east, southeast and northwest [137,154]. Of those, the northeast area (from Finland to Ukraine along the eastern border of the EU) and east area (from Ukraine to Cyprus) may be of particular interest for HPAI virus introduction into the EU, given that this is the main entry route used by dabbling ducks, diving ducks, geese and swans, shorebirds and gulls [154]. Moreover, the northeast and east route fits previous HPAI H5 virus incursions [155]. Wintering grounds for birds migrating through the northeast/east area are located primarily in Western Europe (e.g., Eurasian wigeon [156]). The east area may be more susceptible to cold spells than the northeast area [157], resulting in late winter arrivals of birds in West or Central Europe through the east area. Most migrating birds divide their journey in several flights, interchanged by stopover periods that may be longer in duration than the flying periods [158,159]. Fall migration can take longer than Spring migration with longer stopover times [160]. The timing of migration depends on the annual cycle of the bird, in particular the breeding biology of the species and whether the main moult of feathers is undertaken at or near the breeding or wintering grounds [154]. Migration from the breeding areas starts in summer, while the main migration period for large parts of Europe is within August to December [154]. Novel HPAI H5 viruses have been detected in wild birds from October onwards, first in northeast/east/southeast Europe followed by central/west/southwest Europe [155]. Stopover sites during migration after the breeding period and, in particular, during moult, may facilitate aggregations of different breeding populations for a longer period of time, followed by different southward migration routes (e.g., 22–41 days for greater white-fronted geese (*Anser albifrons*) [161]). High-risk areas of virus introduction along the southern part of the Baltic sea have been identified based on real tracking data of mallards and long-term AIV surveillance in southern Sweden [162]. Mallards in northwest Europe are expected to disperse LPAI viruses over an average distance of 160 km in 3 h, with a maximum distance of 600 km [162]. As the maximum migration distance varies by duck species and individual bird, and by month or season [136,163], the tracking of bird movements became an indispensable tool to further clarify environmental drivers of waterbird migration [164] and aggregations, and to derive predictors for disease dispersal by wild waterbirds.

### 3.5. HPAI H5 Virus Introductions into Poultry Farms by Wild Birds

The risks associated with HPAI virus introduction vary by region, due primarily to differences in poultry production systems (e.g., outdoor farms, farmed mallards to be released for hunting), farm density, and wild bird population distributions. Poultry farming has increased dramatically globally, and, in particular in, Eurasia, in the past 20 years [5]. Open poultry production systems, such as backyard and free-range farms, were often reported as primarily infected farms (e.g., [82]). Based on 24 h video-camera monitoring, 16 different wild bird families were identified visiting the outdoor facility of a farm in the Netherlands that experienced at least six LPAI virus introductions previously [165]. Of the visiting birds, Anatidae (here mallards and unspecified wild duck species) had the longest exposure time to the free-range area from November to February, in particular during January and February [165]. Based on GPS tracking, mallard movement patterns were shown to be highly predictable with regular commuting flights at dusk and dawn [129]. Mallards and other wild duck species (unspecified) visited the farm mainly directly after sunrise and birds would leave before the poultry were let out to roam in the outdoor facility [165]. Therefore, no direct contact between chicken and wild birds was observed, yet ducks were present in the outdoor facility for longer periods of time than other wild bird species. Duck faecal droppings would be directly accessible to free-range poultry, facilitating the exposure of poultry to potential AIV-contaminated faeces.

Seasonal variation in the presence of wild ducks and the vicinity of wetlands were associated with HPAI H5 GsGd virus introductions into poultry farms. A study targeting HPAI H5 host species or species groups (Eurasian wigeon, tufted duck, Anatidae, Laridae) showed that the timing of peak densities of Eurasian wigeons and other Anatidae around HPAI H5 infected poultry farms in the Netherlands (i.e., November–February) coincided with the timing of the HPAI H5N8 outbreaks in poultry, between late November and late December 2016 [166]. Increased wild bird (i.e., Eurasian wigeon, tufted duck, Anatidae, Laridae) counts around poultry farms were much more pronounced for farms located in wetlands than farms not located in wetlands [166], which confirmed earlier findings on distance to open water as a risk factor for HPAI GsGd H5 introduction [167,168]. Indeed, mallard movement patterns are strongly adjusted to the availability of wetlands [129], yet mallards and common teal have also been shown to forage outside wetlands and visit different agricultural fields [169]. Similarly, November to February was identified as the period with the highest risk of virus introductions into poultry, based on LPAI virus introductions into poultry in the Netherlands (2013–2016) [170]. This period covers partly local LPAI virus peak prevalence in mallards (August–December) and in non-mallard ducks (October–December); however, the last group is most abundant locally during winter. In addition, this high-risk period corresponds in time with LPAI virus detections—in particular, of LPAI virus subtypes detected in poultry—in geese (December–February) and swans (December–January) [171]. The time interval between virus introduction and detection was estimated for chicken and duck farms during HPAI H5 virus outbreaks in the Netherlands in 2014 and 2016, resulting in 9.8–14.8 days (chicken 2014), 5.9–7.4 days (chicken 2016), and 9.5–18.8 days (ducks 2016) [172]. Juveniles and migrants have been identified as drivers for seasonal LPAI virus epizootics in mallards [173]. As HPAI viruses appear to be detected after LPAI virus peaks in local mallard populations, additional factors may be of importance in the epidemiology of HPAI viruses at the wild-domestic bird interface, such as the seasonal distribution of waterbirds (e.g., more mallards in croplands during winter than during autumn [174]) and/or the presence of other Anas species [173]. Thus, LPAI and HPAI virus distribution at the wild-domestic interface is shaped by similar risk factors: the presence of wetlands and ducks.

Poultry farms became infected as the result of separate HPAI virus introductions from wild birds, as well as direct from farm-to-farm spread [37,69]. Besides the presence of open water and ducks, the presence of other poultry farms—in particular, duck farms—is associated with increased risk for HPAI H5 GsGd introduction into (gallinaceous) poultry farms in Bulgaria [74]. Additionally, increased risks of AIV transmission were observed in areas of intensive free-ranging duck production with wild waterfowl in south-eastern China [175]. For commercial farms, virus introduction via material contaminated with wild bird material appeared the most likely explanation for the HPAI H5N8 outbreak in Germany in 2014 [176,177]. Introduction of AIVs into poultry farms can occur through, e.g., shared equipment [177,178], and/or contaminated boots and insufficient decontamination footbaths [124]. Groundwater was also reported to play a role in the transmission of AIVs between poultry and wild birds during HPAI H5 virus outbreaks in the midwestern USA [179]. Furthermore, farming practices, such as the disposal of contaminated carcasses in the environment, may have exposed wild birds as well as neighbouring farms to HPAI H5 viruses in Russia [82]. Small birds, such as house sparrows and Eurasian starlings, have been suggested to act as bridge species [180,181]. Indeed, the species are frequently observed at (free-range) poultry farms [165,182] as well as wetlands/cropland [182], a habitat shared with waterbirds. However, no HPAI H5 viruses have been detected in the faecal samples of house sparrows or Eurasian starlings upon natural or experimental virus exposure [183,184,185]. Rodents can be abundant around poultry farms and may play a role in the transmission of HPAI viruses to poultry [186]. Lastly, wind-borne spread may move HPAI H5 viruses, but only over short distances [187].

## 4. Future Directions for HPAI Virus Research and Surveillance

### 4.1. Whole Genome Sequencing

The whole genome sequencing and sharing of sequences are essential to link spatiotemporally separated HPAI H5 virus outbreaks and understand virus epidemiology and evolution [17,18]. Until recently, H5 viruses were predominantly detected and characterized through the partial sequencing of the HA genomic segment. This confirms the HA subtype and genetic clade but lacks information on the gene constellation of the HPAI H5 viruses. Given that HPAI H5 viruses—in particular, of clade 2.3.4.4—have been detected in a myriad of gene constellations, adding complexity to HPAI H5 epidemiology, whole genome sequencing offers higher resolution analytical insight above solely the HA. To understand the formation and spread of these gene constellations, several studies have categorized HPAI H5 viruses into genotypes based on whole genome sequences [20,35,76]. These genotypes are based on publicly available sequences (biased with respect to host species, geographies, and time periods) (e.g., [35,76,107]), yet a universal system quantitatively characterising conditions for HPAI H5 genotype categorization is lacking. We propose that genotype definition could be improved through whole genome phylogenies and incongruence analyses of IAV sequences and their metadata to allow finer grain resolution of evolutionary dynamics and support integrated ecology and evolutionary hypothesis testing. For instance, the reassortment rates of HPAI H5 viruses differ between gulls and ducks [106] highlighting the complex co-infection and diffusion dynamics among different wild bird hosts and emphasising the superficiality of defining "genotype" without integrated and quantitative meta-analyses. Inherently H5 emergence is unpredictable and along with whole genome characterization of HPAI H5 viruses, H5 diagnostics need to be fast, reliable and not too costly in order to act as an early warning system on a large scale. Next-generation sequencing (NGS) techniques are used to characterize whole genome sequences, but these are time consuming, expensive, and require extensive protocols and large stationary equipment. Recent developments in NGS—i.e., third-generation sequencers, including the portable MinION third-generation nanopore sequencing device—facilitate faster, less expensive, easier to use and more mobile analyses of whole viral genomes [188]. A broader application of third-generation sequencing will significantly increase surveillance resolution and efficiency by reducing costs and time, while further optimisation of sequencing techniques and analyses is needed to include minority single nucleotide variant analyses [189]. In addition, to improve methods and technologies, international collaborations and research initiatives and public availability of virus sequence data are crucial in bringing forward the knowledge on HPAI virus emergence, evolution and epidemiology (e.g., The Global Consortium for H5N8 and Related Influenza Viruses, via the GISAID database [17,18,33]). 

### 4.2. Environmental Monitoring

Although evidence-based active surveillance of wild birds is the ideal, environmental sampling sites derived from wild bird migratory patterns may facilitate a less biased approach to AIV surveillance in space and time than the often opportunistic sampling of wild birds. So far, the majority of environmental AIV sampling activities have been focussed on LPAI virus detection and persistence. Indeed, the long-term persistence of LPAI viruses in lakes suggests a role for the environment in LPAI virus maintenance, transmission, and potential for co-infection [87,109,110]. HPAI viruses have been detected with months of no detections in between despite wild bird sampling (e.g., [56,68,134,190]). Whether during these periods of absence, viable HPAI viruses are maintained in the environment and/or by under-sampled or non-sampled bird species deserves further attention. The long-distance transmission of LPAI viruses is likely to be supported by viral persistence in lakes [110,191] and the uptake by migratory birds while stopping over at lakes, continuing the movement of viruses upon deposition in the environment by the previous host(s). Although HPAI viruses may persist in water for a shorter period of time than LPAI viruses [122], the environment may play a role in HPAI virus epidemiology. HPAI viruses have been deposited in the environment by ducks [56,190]. In addition, HPAI H5N1 viruses have been isolated from the digestive tract of wild Mareca and Aythya ducks [139]. Additionally, carcasses of dead birds can contaminate waters used by migratory birds. The chances of HPAI H5 virus detection are low, but with improving virus detection and characterization techniques—such as third generation sequencing—the integration of environmental sampling in routine surveillance programs becomes more feasible [192]. For this, the extraction and detection methods for environmental sampling should be prioritized, further optimised and standardized [3,193]. Furthermore, wild bird observations, in particular, monitoring the mortality of birds of prey (e.g., common buzzard, white-tailed eagle (*Haliaeetus albicilla*), peregrine falcon (*Falco peregrinus*)) could be used as early indicators of HPAI virus incursions.

### 4.3. Host Species Identification

Ducks of the Anas and Mareca genera have been suggested to be vectors of HPAI viruses, yet the majority of wild bird species of the order Anseriformes and Charadriiformes remains unexplored. The capturing and sampling of live wild birds is biased in time and space towards populations of species that are easily accessible. Based on field surveillance, HPAI viruses have been isolated more than once from apparently healthy ducks of the Anas and Mareca genera (Table 2) [56,68,190]. Moreover, these ducks have shown no to minimal clinical signs—despite virus excretion—upon HPAI virus exposure in experimental settings [132,135,139]. Furthermore, HPAI viruses have been isolated more than once from apparently healthy black-headed gulls (e.g., clade 2.2.2 [34] and clade 2.3.4.4.b OIE2016). However, less is known about other species of the order Anseriformes and Charadriiformes; therefore, these should be prioritised within surveillance programs. In addition, the identification of “bridge species” that may transmit HPAI H5 viruses from maintenance populations to domestic birds (“target populations”) [180,181] have been listed previously [138]; however, deserve further attention. Furthermore, HPAI viruses have been detected in a range of wild bird species (Table 2), yet in most cases with unknown health status, whereas the health status of the sampled individual is important to understand the virulence of the different HPAI virus strains. Host species identification should be more structured with a broader, less-biased approach—for instance, host identification based on DNA barcoding of bird droppings, or by serology. Fresh bird droppings collected in a temporarily structured way in wild bird habitats—with or without bird observations—or retrospectively near recently infected poultry farm(s), could be used for both host species identification and the (early) detection of novel HPAI viruses excreted by these hosts. Species can be identified using DNA barcoding that utilises a standardised region of the cytochrome c oxidase subunit I gene (COI) gene to identify specimens to the species level [194,195,196,197]. By using this technique, 66 of 79 (26 genera) of Anatidae species were identified [198]. In addition, targeted next-generation DNA sequencing identified 198 bird species based on genomic sequence data [199,200]. More recently, additional host avian genomes have become available that can be used as reference genomes [201]. The Barcode of Life Database (BoLD) contains sequences referring to more than 5000 of the approximately 10,000 avian species identified globally [202]. The further development of serology would contribute to the identification of bird species acting as vector for HPAI viruses, as these serological data would identify hosts that survive HPAI virus exposure, resulting in seroconversion and have the advantage that fewer birds would need to be sampled compared with virological sampling, owing to longer-term antibody persistence relative to virus shedding [149]. Routinely used AIV-specific blocking ELISA have been shown to be useful to indicate past AIV exposure, although the duration of NP-specific antibody detection may be limited to weeks or months, as demonstrated for LPAI viruses [203]. Haemagglutination inhibition and virus neutralisation tests detected HPAI H5-specific antibodies in ducks at least 34 days upon exposure [152]. Protein micro-arrays are promising alternatives for the “gold-standard” hemagglutination inhibition assays [204,205] but require further optimization. In addition, these wild bird sera can be used to investigate potential antigenic variation of LPAI and HPAI viruses in wild birds [149]. Additionally, the detection of AIV-specific mucosal antibodies in swabs deserves further research, given the gastro-intestinal tract tropism of AIVs in ducks [206]. Better knowledge about bird species that act as HPAI vectors would facilitate the targeted capturing and sampling of wild birds and improve risk assessments. 

### 4.4. Wild Bird Surveillance Site Selection

All host species that can act as vectors for HPAI viruses—with particular focus on migratory Anatidae—should be implemented in wild bird AIV surveillance design, taking into account their migratory behaviour, in order to act as an early warning system. Currently, active wild bird surveillance (i.e., sampling of live birds) within Europe is most intensively performed in northwest Europe in winter (EC reports 2005–2017 [207]), whereas the northeast/east area of Europe serves as access for novel virus introductions both from Asia as well as from/into Africa [208,209,210]. Surveillance programs in wild birds should investigate accessible sites where sufficiently large numbers of birds can be sampled, for all relevant vector species. These sites may reside anywhere in between the breeding and wintering areas, with a preference for sites towards the north and northeast to facilitate earlier detection of novel HPAI H5 viruses upon introduction into Europe. Surveillance should start shortly after the breeding period, perhaps during moult, until the early arrival of each species at the wintering sites in fall. For example, in the case of, e.g., greater white-fronted geese, fall migration stopover sites were identified along the southern coast of the Baltic Sea (e.g., Baltic Sea coast of Estonia, Lake Peipus in Estonia) and in the north-western part of the Black Sea coast in Romania [161]. However, other species may migrate to their wintering sites without stopover periods in easily accessible areas where birds can be sampled and may thus only be targeted for surveillance upon their earliest arrival in Europe. As stopover and moulting sites are less well described than breeding and wintering sites, active wild bird surveillance should be further targeted based on bird GPS tracking movement data and citizen-based bird observation networks [211,212]. 

### 4.5. Active Surveillance and Mitigation

Introductions and outbreaks of HPAI H5 viruses in poultry and wild birds should be monitored closely through existing passive surveillance complemented by active surveillance. Criteria for risk-based surveillance in poultry related to virus introduction (e.g., proximity of water, high wild bird densities) and virus spread within poultry (e.g., poultry farm type, high poultry farm densities) have been listed by the European Commission [207]. In Europe, poultry is routinely sampled for the presence of AIV-specific antibodies [207]. In addition, the use of eggs instead of serum has shown to be a good and easier-to-apply alternative to sera for the early detection of LPAI-specific antibodies [213]. However, in order to detect novel HPAI H5 viruses earlier upon introduction, passive surveillance should be complemented by active surveillance. In poultry, syndromic surveillance in poultry (i.e., decrease food or water uptake, decrease in egg laying) and increased mortality have shown to be strong indicators of HPAI virus infection [79,214]. In wild birds, the passive surveillance of birds found dead should be complemented by the sampling of scientifically-based target species at locations derived from wild bird migration (see Section 4.4). Furthermore, attention should be paid to systemic documentation and the consistent use of terminology, based on findings of a study on active animal surveillance in the EU in 2011 [215].

Currently, a range of recommendations to lower the risk of HPAI virus introductions into farms are available (e.g., [185,216]), yet partly supported by evidence of risk reduction. Evidence-based mitigations determine efficacy (e.g., percentage of risk reduction) and may affect farmers’ motivation to implement the associated measure [217]. Lessons on evidence-based HPAI virus mitigation strategies can be learned from more common poultry pathogens, such as LPAI viruses and Campylobacter [218]. In addition, regions have their own production characteristics and require region-specific mitigation strategies (e.g., [219]). Meanwhile, new poultry farms should not be placed close to other farms, or in/near wild waterbird habitats. Established poultry farms should investigate ways to make the direct farm environment less attractive for waterbirds [220].

## 5. Conclusions

The recurring incursions of HPAI H5 GsGd in Europe (2005—2020) show high viral genetic diversity and can cause severe disease in both domestic and wild birds. Wild birds show variable susceptibility to disease and facilitate the abundant reassortment of genomic segments [20,35,76,221]. The massive growth of the poultry industry globally [5] and the continued circulation of HPAI H5 viruses, in particular, in southeast Asia and northeast Africa [24], combined with the spillback of viruses into migratory birds pose a continuous risk for novel viruses to emerge and be transported over long distances by wild birds. In addition, the recent observed poleward movement of waterbird distributions [127] may drive long-distant virus spread. The HPAI H5 virus outbreaks should be monitored closely, given that a range of wild bird species is susceptible to infection and IAVs are generally unpredictable. So far, human cases due to HPAI H5 viruses have been limited to Asia and Africa. Significant progress has been made in understanding HPAI H5 virus evolution and epidemiology—in particular, with respect to the identification of the host species, time periods, habitats and geographies associated with increased risks of HPAI H5 introduction—due to the collaborative efforts of virologists, ornithologists, ecologists, pathologists, and mathematical modellers, amongst others, and due to faster and specific diagnostics; in particular, gene sequencing technologies. In order to detect novel HPAI H5 viruses early upon introduction into Europe, well-structured, long-term surveillance is essential. Therefore, passive poultry and wild bird surveillance should be integrated with active wild bird surveillance aiming at virus detection in target-species at priority sites along their migratory flyway from their breeding period onwards. Wild and domestic bird surveillance programs need to prioritize wild bird host species identification and incorporate avian ecology to improve the early detection and monitoring of novel HPAI H5 viruses. These long-term surveillance programs serve to better understand ecology and trends in virus prevalence and diversity to be used to work towards prediction of virus incursions in time and space. In addition, these surveillance programs can be used for other wild bird-related emerging pathogens. As long as current agricultural practices are applied, more novel viruses, such as the HPAI H5 virus, will emerge and may spread dramatically, posing a risk for animal and human health globally. Ultimately, the most expedient way to address the global One Health challenge posed by avian influenza is to effectively control poultry disease and support safe and sustainable food production, which in turn would reduce both the threat to wildlife and the zoonotic risk to humans in the long run. Understanding avian influenza ecology and evolution in wild birds is a critical component in any future global One Health framework, which should integrate evidence-based and science-driven improved food security, animal health and socio-economic development with emerging infectious disease mitigation.

## Figures and Tables

**Table 1:** Highly pathogenic avian influenza H5 GsGd virus incursions into Europe, 2005–2020. P, poultry; W, wild birds. Green, viruses detected in wild birds; orange, viruses detected in poultry; light green, clade 2.2 viruses detected in wild birds and subclade unknown; light orange, clade 2.2 viruses detected in poultry and subclade unknown. References used [24,34,35,37,38,39,40,41,42,43,44,45,46,47,48,49,50,51,52,53,54,55,56,57].

Clade	2.2						2.3.2.1				2.3.4.4									
Subclade	2.2.1		2.2.2		2.2		2.3.2.1c		2.3.2.1c		2.3.4.4a		2.3.4.4b		2.3.4.4b		2.3.4.4b		2.3.4.4b	
Period	2005–2006		2005–2006		2006–2009		2010		2015		2014–2015		2016–2019		2017–2019		2019–2020		2020–2021 (ongoing)	
Subtypes identified	H5N1		H5N1		H5N1		H5N1		H5N1		H5N8		H5N5 H5N6 H5N8		H5N6		H5N8		H5N1 H5N3 H5N4 H5N5 H5N8	
Host type	W	P	W	P	W	P	W	P	W	P	W	P	W	P	W	P	W	P	W	P
Country																				
Albania																				
Andorra																				
Armenia																				
Austria																				
Azerbaijan																				
Belarus																				
Belgium																				
Bosnia Herzegovina																				
Bulgaria																				
Croatia																				
Cyprus																				
Czechia																				
Denmark																				
Estonia																				
Finland																				
France																				
Georgia																				
Germany																				
Greece																				
Hungary																				
Iceland																				
Ireland																				
Italy																				
Kosovo																				
Latvia																				
Liechtenstein																				
Lithuania																				
Luxembourg																				
Malta																				
Moldova																				
Monaco																				
Montenegro																				
Netherlands																				
North Macedonia																				
Norway																				
Poland																				
Portugal																				
Romania																				
Russia																				
San Marino																				
Serbia																				
Slovakia																				
Slovenia																				
Spain																				
Sweden																				
Switzerland																				
Turkey																				
Ukraine																				
UK																				
Vatican City																				

**Table 2:** Highly pathogenic avian influenza viruses of the GsGd lineage detected in wild birds in Europe, 2005–2020. Blue, viruses detected in at least one apparently healthy individual of the wild bird species; green, viruses detected in wild bird species; light green, clade 2.2 viruses detected in wild birds and subclade unknown. (•) indicates species–subclade combinations reported in more than one European country. References used [24,34,35,37,38,39,40,41,42,43,44,45,46,47,48,49,50,51,52,53,54,55,56,57].

Clade				2.2			2.3.2.1		2.3.4.4				
Subclade				2.2.1	2.2.2	2.2	2.3.2.1c	2.3.2.1c	2.3.4.4a	2.3.4.4b	2.3.4.4b	2.3.4.4b	2.3.4.4b
Period				2005–2006	2005–2006	2006–2009	2010	2015	2014–2015	2016–2019	2017–2019	2019–2020	2020–2021 (ongoing)
Subtypes identified				H5N1	H5N1	H5N1	H5N1	H5N1	H5N8	H5N5 H5N6 H5N8	H5N6	H5N8	H5N1 H5N3 H5N4 H5N5 H5N8
Order	Family	Species	Latin										
Anseriformes	Anatidae	Graylag goose	*Anser anser*		∙					∙	∙		∙
		Greater white-fronted goose	*Anser albifrons*							∙			∙
		Lesser white-fronted goose	*Anser erythropus*										
		Taiga bean-goose	*Anser fabalis*										•
		Tundra bean-goose	*Anser serrirostris*										
		Pink-footed goose	*Anser brachyrhynchus*										•
		Brant	*Branta bernicla*										•
		Barnacle goose	*Branta leucopsis*										•
		Canada goose	*Branta canadensis*		•								•
		Red-breasted goose	*Branta ruficollis*										
		Mute swan	*Cygnus olor*	•	•	•				•	•		•
		Black swan	*Cygnus atratus*							•			
		Whooper swan	*Cygnus cygnus*		•					•			•
		Tundra swan	*Cygnus columbianus*							•			
		Egyptian goose	*Alopochen aegyptiaca*										
		Ruddy shelduck	*Tadorna ferruginea*										
		Common shelduck	*Tadorna tadorna*							•			•
		Muscovy duck	*Cairina moschata*										
		Northern shoveler	*Spatula clypeata*										
		Gadwall	*Mareca strepera*										
		Eurasian wigeon	*Mareca penelope*						•	•			•
		Mallard	*Anas platyrhynchos*	•						•	•		•
		Northern pintail	*Anas acuta*										
		Eurasian green-winged teal	*Anas crecca*							•			•
		Red-crested pochard	*Netta rufina*										
		Common pochard	*Aythya ferina*	•						•	•		
		Tufted duck	*Aythya fuligula*	•	•					•	•		
		Greater scaup	*Aythya marila*										
		Common eider	*Somateria mollissima*										•
		Common goldeneye	*Bucephala clangula*										
		Smew	*Mergellus albellus*										
		Common Merganser	*Mergus merganser*	•	•								
Galliformes	Phasianidae	Ring-necked Pheasant	*Phasianus colchicus*								•		
Podicpediformes	Podicipedidae	Little grebe	*Tachybaptus ruficollis*										
		Great crested grebe	*Podiceps cristatus*	•		•				•			
		Eared grebe	*Podiceps nigricollis*										
Columbiformes	Columbidae	Rock pigeon	*Columba livia*										
		Common wood-pigeon	*Columba palumbus*										
		Eurasian collared-dove	*Streptopelia decaocto*										
Gruiformes	Rallidae	Eurasian moorhen	*Gallinula chloropus*										
		Eurasian coot	*Fulica atra*	•						•			
	Gruidae	Common crane	*Grus grus*										
Charadriiformes	Haematopodidae	Eurasian oystercatcher	*Haematopus ostralegus*										
	Charadriidae	Northern lapwing	*Vanellus vanellus*										
	Scolopacidae	Eurasian curlew	*Numenius arquata*										•
		Red knot	*Calidris canutus*										•
		Curlew sandpiper	*Calidris ferruginea*										
		Green sandpiper	*Tringa ochropus*										
	Laridae	Black-headed gull	*Chroicocephalus ridibundus*							•	•		•
		Mew gull	*Larus canus*							•			
		Herring gull	*Larus argentatus*							•	•		•
		Armenian gull	*Larus armenicus*										
		Lesser black-backed gull	*Larus fuscus*										
		Great black-backed gull	*Larus marinus*							•	•		
		Common tern	*Sterna hirundo*										
Ciconiiformes	Ciconiidae	White stork	*Ciconia ciconia*							•			
Suliformes	Sulidae	Northern gannet	*Morus bassanus*										
	Phalacrocoracidae	Pygmy cormorant	*Microcarbo pygmaeus*										
		Great cormorant	*Phalacrocorax carbo*							•			•
Pelecaniformes	Pelecanidae	Dalmatian pelican	*Pelecanus crispus*										
Pelecaniformes	Ardeidae	Great bittern	*Botaurus stellaris*										
		Gray heron	*Ardea cinerea*							•			•
		Great egret	*Ardea alba*							•			•
		Little egret	*Egretta garzetta*										
		Cattle egret	*Bubulcus ibis*										
	Threskiornithidae	Eurasian spoonbill	*Platalea leucorodia*										
Accipitriformes	Accipitridae	Hen harrier	*Circus cyaneus*										
		Eurasian sparrowhawk	*Accipiter nisus*										•
		Northern goshawk	*Accipiter gentilis*								•		
		White-tailed eagle	*Haliaeetus albicilla*							•	•		•
		Common buzzard	*Buteo buteo*		•					•	•		•
Strigiformes	Strigidae	Eurasian eagle-owl	*Bubo bubo*										•
		Tawny owl	*Strix aluco*										
		Short-eared owl	*Asio flammeus*										
Falconiformes	Falconidae	Eurasian kestrel	*Falco tinnunculus*							•			
		Saker falcon	*Falco cherrug*										
		Peregrine falcon	*Falco peregrinus*							•			•
Passeriformes	Corvidae	Eurasian magpie	*Pica pica*							•			
		Rook	*Corvus frugilegus*							•			
		Hooded crow	*Corvus cornix*										
		Common raven	*Corvus corax*										
	Sturnidae	European starling	*Sturnus vulgaris*										
	Turdidae	Song thrush	*Turdus philomelos*										
		Eurasian blackbird	*Turdus merula*										
		Fieldfare	*Turdus pilaris*										
	Passeridae	House sparrow	*Passer domesticus*										

## Data Availability

No new data were created or analyzed in this study. Data sharing is not applicable to this article.
